# Remaining life expectancy among older people in a rural area of Vietnam: trends and socioeconomic inequalities during a period of multiple transitions

**DOI:** 10.1186/1471-2458-9-471

**Published:** 2009-12-17

**Authors:** Le V Hoi, Ho D Phuc, Truong V Dung, Nguyen TK Chuc, Lars Lindholm

**Affiliations:** 1 Epidemiology and Public Health Sciences, Department of Public Health and Clinical Medicine, Umea University, S-901 85, Umea, Sweden; 2 Faculty of Public Health, Hanoi Medical University, 01, Ton That Tung Str, Hanoi, Vietnam; 3 Institute of Mathematics, Vietnamese Academy of Science and Technology, 18, Hoang Quoc Viet Rd, Hanoi, Vietnam; 4 Department of Science and Training, Ministry of Health, 138A, Giang Vo Str, Hanoi, Vietnam; 5 Health System Research Project, Vietnam-Swedish Collaborative Sub-Program in Health, Hanoi Medical University, 01, Ton That Tung Str, Hanoi, Vietnam; 6 Ageing and Living Conditions Programme, Centre for Population Studies, Umeå University, S-901 85, Umea, Sweden

## Abstract

**Background:**

Better understanding of the trends and disparities in health at old age in terms of life expectancy will help to provide appropriate responses to the growing needs of health and social care for the older population in the context of limited resources. As a result of rapid economic, demographic and epidemiological changes, the number of people aged 60 and over in Vietnam is increasing rapidly, from 6.7% in 1979 to 9.2% in 2006. Life expectancy at birth has increased but not much are known about changes in old ages. This study assesses the trends and socioeconomic inequalities in RLE at age 60 in a rural area in an effort to highlight this vulnerable group and to anticipate their future health and social needs.

**Methods:**

An abridged life table adjusted for small area data was used to estimate cohort life expectancies at old age and the corresponding 95% confidence intervals from longitudinal data collected by FilaBavi DSS during 1999-2006, which covered 7,668 people at age 60+ with 43,272 person-years, out of a total of 64,053 people with 388,278 person-years. Differences in life expectancy were examined according to socioeconomic factors, including socio-demographic characteristics, wealth, poverty and living arrangements.

**Results:**

Life expectancies at age 60 have increased by approximately one year from the period 1999-2002 to 2003-2006. The increases are observed in both sexes, but are significant among females and relate to improvements among those who belong to the middle and upper household wealth quintiles. However, life expectancy tends to decrease in the most vulnerable groups. There is a wide gap in life expectancy according to poverty status and living arrangements, and the gap by poverty status has widened over the study period. The gender gap in life expectancy is consistent across all socioeconomic groups and tends to be wider amongst the more disadvantaged population.

**Conclusions:**

There is a trend of increasing life expectancy among older people in rural areas of Vietnam. Inequalities in life expectancy exist between socioeconomic groups, especially between different poverty levels and also patterns of living arrangements. These inequalities should be addressed by appropriate social and health policies with stronger targeting of the poorest and most disadvantaged groups.

## Background

The proportion of people aged 60 and older out of the total population in Vietnam has increased remarkably in recent decades, from 6.7% in 1979 to 8.1% in 1999 [1] and 9.2% in 2006 [2]. It has been projected that this age group will grow faster than any other, amounting to 13.4% and 26.1% of the total population in 2025 and 2050, respectively [3]. The increase of older people has been influenced by the current process of multiple transitions in the country.

*Firstly*, Vietnam's economic transition was initiated by the Government's adoption of a wide range of economic-policy reforms in1986 that shifted the country from a central planning economy to a market economy, which led to a strong GDP growth rate, increasing from 3.4% in 1986 to an average of 8% per year from 1992 to 2006 [4-6]. The positive results for economic development have significantly contributed to improved living standards of households [7]. However, inequality in income increased temporary migration from the rural to urban areas because of better employment opportunities [5]. This movement of young people and other impacts of the country's economic transition may have a negative effect by weakening the traditional family structure and leaving more older people to live on their own with less physical and emotional support from family members [8]. On one hand older men are losing the benefits of living in an extended household where they receive more emotional and physical support from the women of the household, but on the other hand older women may benefit as there are fewer expectations and demands for them to do house work and nurture the family in a less extended family.

*Secondly*, along with improved living standards and health care in the last decades during the economic transition in Vietnam, crude mortality rates estimated from population surveys and censuses have decreased from around 10 per 1,000 at the end of the 1970s to 7.5 at the end of the 1980s, 5.6 at the end of the 1990s [9] and fluctuating between 5.6-5.8 in the first five years of the 21^st ^century [10]. The vital registration system in the country does not operating effectively [11]. The system cannot provide completed and accurate data on the number of deaths, cause of death, age, sex, and living standard of people who died. Most of routine figures on death rates are estimated from public hospital data. Therefore, the figures likely under- and misreport deaths. Following the introduction of government policy aimed at lower population growth since the 1970s and then successful implementation of the national family planning programme, fertility has substantially reduced from almost 6 births per woman to the current level of 2.1 births, which is almost equal to the replacement rate [12]. As a consequence, Vietnam's population initiated a rapid aging process, with declines in both fertility and mortality. The decline in fertility is the primary factor responsible for population aging. It directly influences aging at both the population level and the individual level since it increases the level and speed of the aging process in a population and directly influences the number of potential caregivers in the immediate family [8]. The decline in mortality has resulted in a longer life expectancy of the Vietnamese population, with WHO's estimated life expectancy at birth increased from 66 years in 1990 to 70 years in 2000 and 72 years in 2006 [13,14] and are projected to increase to 77.1 years and 80.3 years by 2025 and 2050, respectively [3].

*Thirdly*, an epidemiological transition has been emerging in Vietnam. Incidence of communicable diseases (CDs) has fallen while the incidence of non-communicable diseases (NCDs) has increased in recent decades [5,12]. The contribution of CDs in annual numbers of cases and deaths due to all causes medically diagnosed in public hospitals decreased from 55.5% and 53.1% in 1976, to 24.9% and 13.2% in 2006. The share of NCDs in the total morbidity increased from 39.0% in 1986 to 62.4% in 2006, and in the total mortality from 41.1% to 61.6%, respectively. NCDs are the leading causes of death among both young adults and older people [15], and the incidence of NCDs has increased rapidly with age, especially among elderly people [16]. But deaths in public hospitals are accounted for only about 5% of the total annual mortality and cannot reflect the general mortality patterns of the population [11].

In addition to the above transitions, there have been remarkable changes in the network of caregivers for older people mainly due to social changes aimed at more equal gender roles. These changes have been facilitated by government efforts to encourage new lifestyles for a modern society. In particular wives, daughters and daughters-in-law have experienced changes in their traditional roles from just nurturing their family to paid work outside the home and also changes in their social roles. In terms of social welfare, while older people in the country are less financially reliant on their dependants due to retirement salaries, the rural elderly rely less on social welfare but more on material support from their family.

With such a multi-dimensional change, Vietnam is now faced with new emerging health issues. Elderly health care that has been less of a priority than many health issues in other vulnerable groups (ethnic minorities, children, women and the poor) has now become an important issue. As a basic indicator of population health, increased life expectancy has been set as a key target in national health plans and national socio-economic development plans [5,12,17]. In addition, the increase of life expectancy has been largely defined as a key indicator of successful aging [18]. Therefore, life expectancy at old age can be an appropriate indicator to examine changes in overall health status among older people during the current transitional period of the country.

Higher socioeconomic status (SES) has been associated with better health and longer life in different eras, genders and ages in many other countries, and a variety of health outcomes [19]. Different methods can be used for measuring inequalities in health. There has been a focus on measuring health inequalities between different socioeconomic groups classified by education, ethnicity, income etc. [20]. Death rate and life expectancy are common indicators of a population's health status, and assessment of health inequalities based on life expectancy is useful for health policy and is feasible in small areas [21,22].

While socioeconomic inequalities in health are well documented in the industrialised world [23], literature on health inequalities in low- and middle-income countries is limited, particularly with regard to changes in inequality over time within a country [24,25]. Furthermore, there has been very little research on socioeconomic inequalities in health for older populations in developing countries [25]. Vietnam is not an exception; there is limited evidence of inequalities in health, particularly among older people and also measured using longitudinal data.

Within the current Vietnamese context of rapid socioeconomic development with widening gaps in income and living standards between social groups and areas [17], national health policy has paid much attention to overcoming inequalities in health, in addition to improving the overall health status of the population [5].

In Vietnam, as in most other developing countries, a majority of the population live in rural areas [26], where socioeconomic status is lower and the aging process of the population is faster than in urban areas [27]. In 2006, the total population reached 84 million, of which 72.9% lived in rural areas [28]. The proportion of people aged 60 and over among the rural population increased from 7.4% in 1989 [27] to 9.9% in 1999 [29]. The elderly population in rural areas accounted for 77.7% of total elderly people in 1993 and 73.3% of the total in 2004 [30]. This slight reduction was due to urbanisation in the country, where the rural elderly were disadvantaged in terms of educational attainment, housing quality, access to media, [30], poverty status [29], and access to health care [31].

Since 1999, a demographic and health longitudinal surveillance system called *FilaBavi *has been operating in the rural Bavi district of Vietnam under the International Network of field sites with continuous Demographic Evaluation of Population and Their Health (INDEPTH). General characteristics of the district and field site have been presented elsewhere [32]. The present study has taken advantage of the routine collection of qualified socioeconomic and health data in FilaBavi, and has been conducted to estimate changes in overall health status - in terms of remaining life expectancy (RLE) - among older people in the rural setting throughout the transitional period in the country in the last decade. The specific objectives of the study were to measure the trends of RLE at old age, and to assess socioeconomic inequalities in RLE at age 60 among groups of older people.

## Methods

Bavi district has an area of 410 km^2 ^with lowland, highland and mountainous areas. It is composed of 32 communes with a total population of 235,000 people in 1999 and 262,763 people in 2007. There are 5 main ethnic groups living in the district. The Kinh ethnic group forms the majority (91%) while the rest includes minorities such as Muong, Dao, Tay, Khme and Hoa. In 1999 0.3% of the adult population were illiterate, and about 69% of the population had completed primary school, 21% secondary level, 9% high school and 0.6% higher education. The majority of the population is Buddhist (90%), while the rest are Catholics or a different denomination. Three-quarters of the district's population work in agriculture.

The longitudinal surveillance system consists of a random sample of 67 out of 352 population clusters in the district. In total, 11,089 households with 51,024 individuals were initially included, accounting for approximately 20% of the total district population in 1999, and approximately equal to a required sample size of 11,000 households for the system. A household baseline survey was conducted at the beginning of 1999 and subsequently every second year [32]. Out of all households followed up by the system, an average of 12,540 households participated in each survey.

Household and individual characteristics of all persons at age 60 and over during 1999-2006 have been extracted from *FilaBavi*'s surveillance database. Variables for individual characteristics include date of birth, death and migration, sex, level of educational attainment, and relationship with the household head and other household members.

Household characteristics include land area, housing structural components, assets, sanitation conditions, income, expenditure and debt. Household assets were classified by certain categories, such as furniture, communication and electricity equipment, types of vehicles, agricultural machines, cattle and others. Assets were classified as "present or not", regardless of the quantity and quality of each item. Sanitation conditions were assessed in terms of water sources for drinking and cooking, type of latrine and presence of a bathroom.

All types of income (agriculture, breeding, forestry and others) were recorded to provide the total income of a given household. The sum of daily food expenditure was multiplied by 30 days and added to the sum of other monthly expenditure to estimate total monthly household expenditure.

An abridged life table constructed according to Chiang's revised methodology [33] has been used internationally to calculate RLE and its confidence intervals. However, the original life table did not take into account the variance of the final age interval, therefore to address this the life table was further adjusted by ONS [34] using the Silcocks method [35] to calculate standard error of life expectancy. The present study used the adjusted life table with age intervals of 5 years to 85+ as an appropriate option for estimating life expectancy in small populations [34,36].

Zero death counts are frequently present at age intervals in small populations. In the adjusted life table, the counts are no longer thought to underestimate standard errors of life expectancy at age intervals, except at the final interval [34]. Thus, a substitution for zero death by using number of deaths estimated from an appropriate national, regional, or locally derived age- and sex-specific mortality rate has been evaluated as an appropriate alternative [36]. In the current study, zero deaths existed only in two socioeconomic groups with the smallest population sizes, including ethnic minorities and females with secondary or higher education. The substitution was made for zero deaths at the final age interval based on sex-specific mortality rates among those 85+ calculated from *Filabavi*'s data collected during 1999-2006.

Life expectancy can be obtained from life tables calculated from period or cohort age-specific mortality rates [37]. The period mortality rate is based on deaths occurred and exposure time spent within a specific age interval over a period of observation. The cohort mortality rate is based on following up people being at a specific age at the beginning of the observation period. Mortality rates are calculated by dividing the number of deaths of these people by the person-time lived by them during the observation period. Additional file 1: Figure A1 illustrates the two ways of calculating age-specific death rates and life tables. In this study estimates of RLE are based on cohort age-specific mortality rates.

In this study, cohort life expectancy and corresponding 95% confidence intervals were estimated using longitudinal mortality data collected in *FilaBavi *during 1999-2006 for groups of older people classified by socio-demographic factors, economic status and living arrangement. Life expectancy was calculated for specific periods (1999-2002, 2003-2006 and 1999-2006) instead of annual estimations in order to maximise the possibility of identifying the significance of any differences between the groups.

Wealth index was measured to assess the economic status of older people's households on the basis that wealth is an underlying unobservable measure relating to relative economic position within a social hierarchy [38]. The location of a particular household within the hierarchy can be assessed through its basic assets and structural components [39]. Household wealth is more suitable than income or consumption [39], particularly among the rural elderly in developing countries, who usually do not earn income and rely more on their families for material survival [38].

Data on household characteristics collected from the baseline survey (1999) and three re-census surveys (2001, 2003 and 2005) were used to calculate the household wealth index. Before the computation, all categorical variables were dichotomised, the continuous economic variables were divided by the number of persons per household to form "per capita" variables, and missing values were replaced by mean values. These missing values are present in data on income (8 variables), expenditure (2 variables) and land/floor areas (3 variables). However, the percentage of observations with missing values per one variable is very low, ranging from only 0.02% to 0.15% among income variables, and from 0.01% to 0.35% among the other variables.

A food poverty line of monthly minimum expenditure required to deliver a daily calorie intake of 2,100 calories per capita has been widely applied to classify household poverty status in developing countries. The food poverty line is added to minimum expenditure for non-food basic needs to form a total poverty line, which is an internationally comparative basic needs poverty line [40]. The current study used an estimate of the total poverty line based on data from the 1998 Vietnam Living Standard Survey - which was equivalent to a monthly expenditure of VND 149,156 (US$ 10.7) per capita [41] - as an international poverty line (IPL).

Specific national poverty lines were also used to classify household poverty status. The level of national poverty lines was affected by the availability of resources for special assistance programmes for the poor [41]. National poverty lines for rural areas based on monthly per capita income were VND 70,000 (US$ 5.0) for 1996-2000 and VND 100,000 (US$ 6.7) for 2001-2005 [42]. All estimates of monthly income or expenditure used for classification of households by a poverty line were further adjusted for inflation using annual exchange rates between the Vietnamese currency and US dollars during 1999-2006.

Across the different study periods older people were classified by socioeconomic variables such as educational attainment, status of household head, the presence of a spouse or grand-/children (sons, sons-in-law, daughters, daughters-in-law, grandchildren) in the household, household wealth index quintiles and poverty lines. The first *three *variables are completely (e.g. ethnicity) or most likely (e.g. residency and education) unchanged over time. The first value measured in a particular period was used for classification during that period.

The remaining socioeconomic variables are more likely to vary over time. All older people identified as household heads from at least one survey during a particular period were classified as household heads during that total period, as were individuals classified as living with a spouse. Living with grand-/children is a less stable variable because of movement or migration among young adults due to marriage, study or employment. Therefore, during a particular period, only those older people identified as living with grand-/children at all surveys and under follow-up were classified as living with grand-/children. The other group includes those living without grand-/children at all surveys.

Households with and without older people were classified into wealth index quintiles for a particular period based on the average value of wealth indices calculated separately from the data of all the surveys during the period. Households were stratified into two poverty status groups according to the poverty line. The first group included households identified as living below a poverty line at all surveys when the older people were alive and under follow-up in a particular period. Households with older people that lived above the poverty line at any survey during the period of follow-up belonged to the second group.

Number of deaths and person-years in different study groups of people aged 60 and over were measured. Percentages of older people in the general population at the baseline survey and re-surveys were also calculated. Distribution of older people by different socioeconomic groups at the surveys was assessed and the corresponding 95% confidence intervals were used for comparison between groups.

95% confidence intervals of RLEs at age 60 were estimated in order to examine significant differences in RLEs between study periods and socioeconomic groups. Trends in RLEs were observed between the two periods of 4 years during 1999-2006, and in all socioeconomic groups. Only the socioeconomic determinants that are significant for mortality, in a stepwise multivariate analysis with Cox regression are considered in the analysis of disparities in RLE (Additional file 1: Table A1). Gaps in RLEs between the groups were examined in terms of absolute difference between their RLEs. The 95% confidence intervals of the gaps were further calculated for comparison between periods or socioeconomic groups.

Ethical approval for the demographic surveillance system of *FilaBavi*, including data collection on vital and socioeconomic statistics, was given by the Research Ethics Committee at Umea University, Sweden (reference number 02-420).

## Results

During 1999-2006 the study covered 7,668 people at age 60 and over with 43,272 person-years (15,941 for male and 27,331 for female), out of a total of 64,053 people with 388,278 person-years followed up by *FilaBavi*. There were 1,399 deaths among the older people during the whole study period. Lengths of follow-up and death counts among the older people by socioeconomic groups in different study periods are presented in Additional file 1: Table A2-4. The profile of the older people at age 60+among the general population at 4 surveys during the study period is described in Additional file 1: Table A5. There is a notable trend of an increased proportion of older people for both sexes.

Distribution of people aged 60+ by socioeconomic factors is presented in Additional file 1: Table A6. Females account for approximately *two-thirds*. At the baseline survey, *one-third *of males had reached the educational level of secondary or higher, while almost all females attained lower educational levels. Education levels for both sexes increased significantly in the next surveys.

A majority of older people are household heads (around 80% for males and 70% for females). *Two-thirds *of females live without a spouse, a figure that remains unchanged between surveys. Only 28.4% of males live without a spouse at the baseline survey, and the proportion of males living without a spouse in the last two surveys reduces significantly compared with previous surveys. Around one-quarter of people of both sexes live without grand-/children and the percentage increases over time.

The percentage of males in the middle to richest quintiles are higher than in the others, whilst females are more equally distributed between wealth quintiles (app 20% in each). The share of males living above the national poverty line is higher than that of females (80 and 72% respectively). The percentage of people living below the international poverty line is higher than those living below the national one. Furthermore, whilst the percentage of people living below the national line is getting lower, the percentage living below the international line reached a peak of more than 50% between 2001 and 2003.

RLE at age 60 for both sexes during 1999-2006 is shown in Table 1. Females can expect to live approximately *seven *years longer than males (26 vs. 19). Comparing the four-year periods, RLE for both sexes increases by approximately *one *year, but the change is only significant for females. RLE for females is significantly higher than for males among all socioeconomic groups during the 8-year period. The gender gap within socioeconomic groups tends to be narrower among the more advantaged population, but only significant between those living with a spouse, and between the poorest and the middle to richest.

**Table 1:** RLE at age 60 and gender gaps by periods and socioeconomic factors, 1999-2006

Sexes/Periods or groups	Male		Female		Gender gap	
	RLE	95%CI	RLE	95%CI	RLE	95%CI
*Periods:*						
1999-2002	18.8	17.9 - 19.6	25.2	24.4 - 25.9	6.4	5.3 - 7.6
2003-2006	19.7	18.8 - 20.5	26.9	26.2 - 27.6	7.2	6.1 - 8.4
1999-2006	19.2	18.6 - 19.8	26.1	25.5 - 26.6	6.9	6.1 - 7.7

*Education*						
Primary or less	18.4	17.6 - 19.3	26.2	25.7 - 26.8	7.8	6.8 - 8.8
Secondary or higher	20.4	19.3 - 21.4	25.2	22.9 - 27.4	4.8	2.4 - 7.3

*Household head status*						
No	15.7	14.2 - 17.1	22.0	20.9 - 23.1	6.3	4.5 - 8.1
Yes	19.7	19.0 - 20.3	26.7	26.2 - 27.3	7.0	6.2 - 7.9

*Living with spouse*						
No	13.5	11.8 - 15.2	25.7	24.9 - 26.4	12.2	10.4 - 14.0
Yes	21.4	20.6 - 22.2	27.4	26.5 - 28.3	6.0	4.8 - 7.2

*Living with grand-/children*						
No	18.5	16.9 - 20.0	26.8	25.4 - 28.1	8.3	6.2 - 10.4
Yes	18.4	17.7 - 19.2	25.0	24.4 - 25.6	6.6	5.6 - 7.5

*Wealth index quintiles*						
Poorest	15.7	14.0 - 17.5	26.1	24.9 - 27.4	10.4	8.2 - 12.5
Poorer	17.0	15.7 - 18.3	25.4	24.2 - 26.6	8.4	6.6 - 10.2
Middle	19.5	18.1 - 20.8	25.1	23.9 - 26.2	5.6	3.8 - 7.3
Richer	20.8	19.6 - 21.9	27.0	25.9 - 28.1	6.2	4.7 - 7.8
Richest	20.9	19.5 - 22.2	26.6	25.3 - 27.9	5.7	3.9 - 7.6

*National poverty line*						
Under	7.6	5.3 - 9.9	15.4	12.9 - 17.9	7.8	4.4 - 11.3
Beyond	20.0	19.4 - 20.6	26.9	26.4 - 27.5	6.9	6.1 - 7.8

*International poverty line*						
Under	12.6	10.8 - 14.3	21.2	19.5 - 23.0	8.6	6.2 - 11.2
Beyond	20.1	19.4 - 20.7	26.7	26.2 - 27.3	6.6	5.8 - 7.5

Higher educational level is associated with longer RLE at a borderline significance among males (Table 1). There is no significant difference in RLE by educational levels among females. Between the study periods, RLE for females with higher educational attainment levels decreased by approximately 5 years, however this was not found to be significant. Older people who are household heads have higher RLE (approximately 4 years for males and 5 years for females) compared with those who are household members (Table 1). Comparing the two periods, RLE increases for both sexes regardless of the status of household heads, however this was not found to be significant (Table 2).

**Table 2:** Changes in RLE at age 60 between periods (1999-2002 vs. 2003-2006)

Sexes/Periods or groups	1999 - 2002		2003 - 2006		Change in RLE
	RLE	95%CI	RLE	95%CI	
*Male*					
Primary or less	18.0	16.9 - 19.2	19.0	17.6 - 20.3	1.0
Secondary or higher	20.1	18.6 - 21.5	21.0	19.4 - 22.5	0.9
*Female*					
Primary or less	25.6	24.8 - 26.3	26.9	26.1 - 27.7	1.3
Secondary or higher	28.7	25.0 - 32.4	23.9	21.3 - 26.5	-4.8

*Male*					
Household member	16.0	14.0 - 18.0	16.5	15.0 - 18.1	0.5
Household head	19.2	18.3 - 20.1	20.2	19.3 - 21.2	1.0
*Female*					
Household member	21.0	19.7 - 22.4	22.7	21.5 - 24.0	1.7
Household head	26.1	25.2 - 26.9	27.4	26.6 - 28.3	1.3

*Male*					
Without spouse	11.8	10.1 - 13.5	16.2	13.6 - 18.7	4.4*
With spouse	24.5	23.1 - 26.0	20.4	19.4 - 21.5	-4.1*
*Female*					
Without spouse	24.8	23.8 - 25.8	26.3	25.3 - 27.3	1.5
With spouse	26.5	25.1 - 27.8	31.8	30.2 - 33.4	5.3*

*Male*					
Without grand-/children	18.8	16.7 - 20.9	16.5	15.0 - 18.1	-2.3
With grand-/children	18.4	17.4 - 19.3	19.1	18.1 - 20.1	0.7
*Female*					
Without grand-/children	28.0	26.2 - 29.8	22.7	21.5 - 24.0	-5.3*
With grand-/children	24.2	23.3 - 25.0	25.7	24.9 - 26.6	1.5

*Male*					
Poorest	14.5	12.5 - 16.5	17.6	15.2 - 20.0	3.1
Poorer	16.4	14.5 - 18.4	17.5	15.3 - 19.6	1.1
Middle	19.2	17.3 - 21.2	19.4	17.8 - 21.0	0.2
Richer	20.6	19.0 - 22.2	20.6	18.9 - 22.2	0.0
Richest	21.1	19.2 - 23.0	21.9	19.9 - 24.0	0.8
*Female*					
Poorest	25.2	23.4 - 27.0	26.3	24.1 - 28.4	1.1
Poorer	25.4	23.6 - 27.3	25.3	23.8 - 26.7	-0.1
Middle	22.9	21.4 - 24.3	27.4	25.9 - 28.9	4.5*
Richer	26.1	24.5 - 27.7	26.6	25.1 - 28.2	0.5
Richest	27.5	25.6 - 29.3	28.5	26.6 - 30.5	1.0

*Male*					
Under NPL	13.3	11.0 - 15.7	9.7	7.2 - 12.2	-3.6
Beyond NPL	19.6	18.7 - 20.4	20.7	19.8 - 21.6	1.1
*Female*					
Under NPL	22.8	20.9 - 24.8	20.5	18.0 - 23.0	-2.3
Beyond NPL	25.7	24.9 - 26.5	27.7	27.0 - 28.5	2.0*

*Male*					
Under IPL	15.9	14.2 - 17.7	16.0	14.5 - 17.5	0.1
Beyond IPL	19.5	18.5 - 20.4	21.3	20.2 - 22.3	1.8
*Female*					
Under IPL	24.7	23.2 - 26.3	23.1	21.7 - 24.5	-1.6
Beyond IPL	25.3	24.5 - 26.2	28.9	28.0 - 29.8	3.6*

* Significant difference in the gaps between the two 4-year periods

RLE for males in the poorest and poorer quintiles is significantly lower than those belonging to the next quintile (Table 1). RLE for females varies insignificantly between wealth quintiles. Comparing the different periods, the greatest improvement in RLE for both sexes is among the wealth groups with lowest RLE, but it is only significant for males. The pattern of significant differences in RLE between the wealth groups of males in the first 4-year period is similar to that of the 8-year period. But there is only one significant difference in RLE between the wealth groups of males observed in the latter 4-year period, which is between the poorer and the richest.

RLE is significantly higher - by approximately 12 years - for both sexes among those living above the national poverty line (Table 1). Comparing the two periods, RLE increases among those living above the line, but only significantly for females (Table 2). RLE decreases among all those living below the line, but this was not found to be significant. Older people living above the international poverty line can expect to live significantly longer, (approximately 7 years for males and 6 years for females) than those living below the line (Table 1).

Table 3 presents the gaps in RLE between poverty levels. There is a trend for the gaps to widen between poverty levels against both the national and international poverty lines when comparing the two periods. However the only significant change is against the international line among females. The gaps against the national line tend to be wider than those against the international line, but the difference is only significant for males during 2003-2006.

**Table 3:** Changing gaps in RLE at age 60 between poverty levels

Sexes/Poverty lines	1999 - 2002		2003 - 2006	
	RLE	95%CI	RLE	95%CI
*Male*				
National	6.2	3.7 - 8.7	11.0	8.3 - 13.7
International	3.6	1.6 - 5.5	5.3	3.5 - 7.1
*Female*				
National	2.9	0.8 - 5.0	7.2	4.6 - 9.9
International	0.6	-1.2 - 2.4	5.8	4.2 - 7.5

RLE is significantly longer among people living with a spouse (approximately 8 years for males and 2 years for females) compared with those living without (Table 1). Comparing the study periods, females living with a spouse have significantly higher RLE in the latter period (Table 2). RLE increases significantly by 4.4 years for males living without a spouse and decreases by 4.1 years among those living with a spouse. It is notable that the difference in RLE from living with a spouse tends to decrease over time.

Loss of spouse is illustrated in Table 4. Those who lived without a spouse during 2003-2006 are stratified according to whether they lived with or without a spouse during 1999-2002. It is clearly worse to lose a spouse than to live without for the entire period.

**Table 4:** RLE among older people living without a spouse during 2003-2006 stratified by status of living with a spouse during 1999-2002

Sexes/Status during 1999-2002	Male		Female	
	RLE	95%CI	RLE	95%CI
Without spouse	19.6	15.8 - 23.5	26.5	25.5 - 27.5
With spouse	12.8	9.5 - 16.1	22.7	19.3 - 26.1

* Significant difference in the gaps between the two 4-year periods

During the whole study period (1999-2006) no significant difference in RLE at old age was observed for either sex when living with or without grand-/children (Table 1). However, during the first 4-year period, RLE among females living without grand-/children is significantly higher than among their counterparts (Table 2). In contrast, during the latter period, RLE among older people living with grand-/children is significantly higher (borderline level for males and higher for females) than their counterparts. Furthermore, between the 4-year periods, RLE increases insignificantly among those living with grand-/children but decreases significantly among those living without.

Table 5 shows RLE for different combinations of living arrangements. The worst combination for a male is to live without a spouse and grand-/children. RLE among females living with both a spouse and grand-/children is significantly higher than those living with only a spouse or grand-/children. When living with a spouse, RLE is significantly higher among females who also live with grand-/children. Among those living above the national poverty line, females living without grand-/children have significantly higher RLE than those living with grand-/children.

**Table 5:** RLE at age 60 living with and without grand-/children and a spouse, and also by national poverty line, 1999-2006

Sexes/Periods or groups	Male		Female	
	RLE	95%CI	RLE	95%CI
*Without spouse:*				
Without grand-/children	7.8	5.0 - 10.7	27.1	25.2 - 29.0
With grand-/children	14.1	12.2 - 16.0	24.7	23.9 - 25.6
*With spouse:*				
Without grand-/children	21.7	19.7 - 23.7	24.0	22.3 - 25.7
With grand-/children	20.6	19.6 - 21.5	27.1	25.8 - 28.3
*Living under NPL:*				
Without grand-/children	4.6	1.1 - 8.1	13.2	6.6 - 19.8
With grand-/children	7.7	5.1 - 10.2	11.4	7.8 - 14.9
*Living beyond NPL:*				
Without grand-/children	20.6	18.8 - 22.4	31.1	29.4 - 32.8
With grand-/children	19.0	18.2 - 19.7	25.6	25.0 - 26.3

* Significant difference in the gaps between the two 4-year periods

## Discussion

There is a noticeable trend of increased RLE over time among the rural older people in Bavi. A significant increase in RLE is generally present in females, and particularly so in some socioeconomic groups, such as the non-poor, the middle wealth quintile and those living with a spouse. Among males, a significant increase over time only occurs among those living without a spouse, while there is a significant decrease among those living with a spouse, thus levelling out the large gap to some extent. Inequalities in RLE exist for both sexes with remarkable advantages among household heads, persons living with a spouse and those living above the poverty lines. Some advantages in RLE are present solely among males with secondary or higher education and those in the middle and better wealth quintiles. Females can expect to live significantly longer than males in all periods and all socioeconomic groups.

The majority of available RLE figures is based on period estimation. To our knowledge, the current study is one of the few available cohort calculations of life expectancy at old ages and likely the first in Vietnam. The cohort life expectancy for males at age 60 during 2003-2006 is roughly equal to the situation in the United Kingdom two decades ago, with 19.6 years in 1987 and 15.8 years in 1990 [43]. Life expectancy for Vietnamese females matched more recent levels in this developed country (26.9 years in 2000). Although the estimates from this study are not comparable with measures for the country in terms of time and area scales, the comparison shows a marked increase in RLE among rural older people, particularly for females.

This trend may not be surprising considering the dramatic improvement in levels of population health compared with the country's economic development, which has been documented using other aggregate health status indicators such as infant mortality rate [44,45] and life expectancy at birth [11,46]. Within a context of rapid socioeconomic development, the high level of RLE for females that is fairly comparable with that in developed countries is reasonable, since the difference in ageing among women in countries at varying economic levels is caused by different mortality rates between birth and middle-age rather than that at old age [47]. Strikingly high life expectancy has been documented for other developing countries where per capita wealth and health expenditure are low by international standards [48]. The current study provides additional evidence on Vietnam's exceptional population health, which might be partially explained by the appropriateness of the current health system that has been consolidated and expanded towards achieving the parallel goals of equity and efficiency during rapid development [11]. On the other hand, existing public and private resources for health and elderly care amount to a very small fraction of what is available in Europe, therefore resource levels are not a particularly good predictor for elderly life expectancy.

It has been suggested that socioeconomic variations are the most fundamental causes of inequalities in health [49]. This relationship is also persistent in life expectancy at old age with a tendency of increased longevity in better socioeconomic groups in both developed and developing countries [50-54]. In the same vein, the present study shows that RLE is significantly higher in better economic groups, classified by thresholds of per capita income or expenditure according to the national or international poverty lines. In relation to living conditions defined by household wealth, RLE is significantly higher among males from middle and higher groups than those from the lower groups.

Moreover, the above trend indicates that disparities in life expectancy are influenced more by income and expenditure than by living conditions. It is notable that older people with disadvantaged life expectancy belong to households living below poverty lines at any survey during the follow-up period, which implies that long-term lower economic status causes this disadvantage rather than short-term conditions. The disadvantages in RLE between the poverty levels are very high, as opposed to the relatively weak socioeconomic gradient measured by the wealth index. This shows the effects of extreme poverty on health compared with the prevailing view of a social gradient.

During all periods, inequalities in RLE for males exist between particular wealth quintiles while there is no significant difference in RLE for females across the standard levels. Therefore, it seems that either better living conditions are more beneficial for RLE for males than for females or that the household's economic level reflects well-being of its male members better than that of its female members. Significant difference between the quintiles in the second 4-year period is only observed between the poorer and the richest, while some other significant differences between the quintiles exist in the first period. This indicates a reduced inequality in RLE caused by living conditions, which can probably be explained by the overall improvement of household living standards during the country's economic reforms.

The gap in RLE against the national poverty line tends to be wider than the international one This indicates that the threshold set for the national line in terms of per capita income level, which is based on the availability of government resources to support the poor, might be lower than necessary for older people to reach a health status that is achievable with the minimum expenditure in order for people's basic needs to be met. While people living below the poverty lines face a lower life expectancy between the four-year periods, there is a trend of increased RLE among those living above the poverty lines. This shows that poverty burden on life expectancy is heavier among those living below the lines, although socioeconomic conditions in society are generally improved. Furthermore, the gap in RLE between different poverty status widened across the four-year periods, which indicates a greater influence of economic disparities on longevity inequalities over time.

The highest gains in RLE are among the wealth groups with lowest RLE in the first period, thus reducing the disparity between wealth quintiles. This change might be attributed to investments in equitable social policies targeted to the poor and other vulnerable groups since 1998 [41,55,56]. These large-scale investments include support for improving commune infrastructure (electricity supply, construction of road, school and commune health stations) in the most disadvantaged areas, and implementation of extensive public health programmes, including maternal health care and free access to health care for the poor. Nevertheless, the gain in RLE among males in the poorest quintile is not large and homogenous enough to be significant. This suggests a need for additional support to enable improvement in health status at old age below the poorest living conditions in society.

In Vietnam, as in many developing countries, family is the main source of financial and material support for older people rather than the state [57-59]. Mutual exchanges exist, however, with older people typically contributing in different ways to support the well-being of other family members [57,60]. A partner is one of the most important sources of emotional and practical support [61]. This is confirmed in the present study where living with a spouse guarantees a significantly higher RLE for both sexes. This shows important mutual support between spouses that is beneficial for better longevity. Furthermore, the transition from living with a spouse to without implies a health risk. Those experiencing this transition have a lower RLE than those who have lived without a spouse in both periods; the loss of a spouse through death or divorce usually implies major grief, stress and worry as well as the loss of social and material support [61].

RLE gained from living with a spouse is higher among males than females. This suggests that males benefit more by living with a spouse than females. This gain in RLE for males might result from the traditional role of women in nurturing their family. The tendency for a decreased difference in gained RLE over time from living with a spouse indicates the affect of changing social roles of women during recent decades.

On initial examination in the current study, living with grand-/children seems to be neutral for disparities in RLE. However, the influence of living with grand-/children becomes evident when stratified by periods, or adjusted for living with a spouse and poverty status. Particularly among older people living without a spouse, males benefit in RLE from living with grand-/children while females conversely so. The benefit for males might result from the patrilineality and patrilocality [62] which are still strong in rural Vietnam. The lower RLE for females when living with grand-/children might be due to the common feature in rural areas of developing countries where older women are involved in domestic work, including caretaking of younger children [57].

When living with a spouse, there is no significant difference in RLE for males, regardless of whether they also live with grand-/children or not. This suggests that daily material and emotional support for older men from their spouse is essential and more important for longevity than support from only grand-/children. Females who live with a spouse gain in life expectancy when also living with grand-/children. The gain might partially result from sharing the parental burden of care for their children with their husband.

When living above the national poverty line, RLE is significantly higher among older women living without grand-/children than those living with. This might be due to the lifestyle practices suitable for old ages, which are different from those of younger generations, as well as from a reduction in domestic workload and childcare. However, the tendency for a higher RLE over time among older people living with grand-/children suggests that the parental role changes with time.

Finally, yet importantly, in terms of living arrangements, RLE is significantly higher for both sexes among household heads than among household members. This shows the important role of household decision-making in improving health status among older people, particularly relating to household health expenditure and food consumption.

Education is one of the key socioeconomic channels for acting on inequalities in health, since it shapes occupational opportunities and earning potential [49] which consequently affect living standards and health care. Education affects disparities in life expectancy until old age because health status at a young age still influences the expectancy at the later ages [63]. In addition, education also provides basic knowledge and life skills that enable better-educated people to gain more ready access to information and resources to promote health during their whole lifespan [64]. Educational disparity in life expectancy also exists in the present study, where RLE is significantly higher among males with better education. This finding, although limited to men, is consistent with studies in other countries with different development levels [54,63,65]. A wide confidence interval of RLE resulting from a very small proportion of females with better education might hide potential inequalities in educational levels among females.

The gender gap in RLE exists consistently among socioeconomic groups, but varies insignificantly. One hypothesis is that the gender gap is less dependent on socioeconomic status and living conditions and more on inherent internal biological factors. A second hypothesis is that the gap can be attributed to different ways of living. Femininity is in many societies associated with carefulness and caution, which may protect against health-damaging lifestyles. Masculinity, on the other hand, may be demonstrated by risky behaviour such as smoking, drinking, high speed driving, which is most pronounced among the lowest social classes. Typical male occupations may also be more risky. The latter hypothesis thus means that the gender gap in old ages is a consequence of accumulated variations in risk exposure during the whole lifespan, which this material gives some support for.

The gender gap is also influenced by the existence of a spouse and is twice as influential in the group living without a spouse than those living with. This figure is shaped by the larger beneficial effect for men of living with a spouse. The reduced gap in better wealth quintiles suggests that improvement of household wealth can narrow the gap in the rural setting, although it is more beneficial for males than for females.

Mortality data collected by *FilaBavi *has been evaluated as an accurate source able to identify 99.8% of deaths in communities from the quarterly household follow-up surveys and 96.0% from the re-census. Official data documented by the Community Registration System (CRS), however, missed 19% of deaths with the majority amongst infants and the elderly [66]. Among deaths at age 60+, the re-census missed 3% for both sex and CRS missed 13% for males and 19% for females. This small gap in data from FilaBavi might therefore lead to a slight overestimation of RLE at old age.

The older persons under the current study were all born before 1946 when a birth registration system did not exist in rural Vietnam. The official registrations of birth data were introduced a couple of decades later and thus there may be some recall bias in the data, especially from the oldest people. Consequently, calculation of their ages might be affected by the bias, which affected the estimation of life expectancy in the study. It has been indicated in other studies that age overstatement is a source of bias in mortality rates at older ages [67]. A formal check of possible age overstatement by using a comparison between T80/T60 in *FilaBavi *DSS data and a "golden standard" country (e.g. Sweden) has been performed. It shows that there is no reason to suspect age overstatement in the data (Additional file 1: Table A7).

Socioeconomic data used for analysis in this study were extracted mainly from four surveys that were conducted every second year. The present study did not account for possible temporary or sudden shifts in socioeconomic status, such as income, expenditure and living arrangements, which might occur between the surveys.

The use of means for missing values of continuous variables of income, expenditure and land/floor area in calculating the wealth index might cause a misclassification of household wealth toward the middle quintile. However, the fact that the percentage of observations with missing values is very low might not distort this index estimate significantly as it that takes into account many other variables.

Currently, few multivariate analysis methods exist for life expectancy studies, especially for small-area data. The present study mainly used univariate analysis methods for examining a single factor's influence on life expectancy at old age. Therefore, most interactions between socioeconomic factors have yet to be considered. Only for a few factors - such as living with grand-/children, which did not show significant disparities in univariate analysis - have the relevant socioeconomic groups been further stratified by certain other factors to consider possible interactions.

There are some skews in distribution of older people across particular socioeconomic factors, which implies a small sample size in some groups such as females with higher educational levels. This might reduce the possibility of identifying significant disparities in RLE related to these small groups. Furthermore, changes in life expectancy usually need to be observed on a larger scale for a long period. Initial results from the present study have been obtained from a small area after only a short follow-up time. Some changes between particular socioeconomic status may not have been recorded.

## Conclusions

Life expectancy at older age in rural area has reached a high level comparable to that of developed countries, particularly for females. Life expectancy increases between the two periods during 1999-2006; this increase is significantly attributed to the improvement among females and the less poor groups. However, life expectancy tends to decrease in the most vulnerable groups, such as people living below the poverty lines and those living without grand-/children. Furthermore, there are wide gaps in RLE between different poverty levels and also living arrangements. The gap in RLE by poverty status is getting wider over time. A gender gap exists consistently in all socioeconomic groups and tends to be wider amongst the more disadvantaged groups.

The notable increase in life expectancy at old age confirms the appropriateness and effectiveness of current social and health policies targeted at older people in Vietnam. These policies should be implemented more extensively. Furthermore, the existing gaps in remaining life expectancy suggest that the policies and corresponding interventions should be more strongly focused on the poorest and most disadvantaged groups with additional support at more appropriate levels. Studies on trends and disparities in life expectancy among older people in Vietnam should be extended to cover different settings and for longer periods in order to provide comprehensive evidence necessary to design appropriate policies under rapid multiple transitions in the country.

## Competing interests

The authors declare that they have no competing interests.

## Authors' contributions

LVH conceived and designed the research, developed the statistical analysis strategies, participated in the data analysis and drafted the manuscript. HDP participated in developing the analysis strategies, performed the statistical analysis, and participated in drafting some sections of the manuscript. TVD revised the research design and the manuscript. NTKC helped with data acquisition and revised the manuscript. LL helped during the conception phase, advised on the research design and data analysis, and revised the draft manuscript. All authors read and approved the final manuscript.

## Pre-publication history

The pre-publication history for this paper can be accessed here:

http://www.biomedcentral.com/1471-2458/9/471/prepub

## Supplementary Material

Additional file 1 **Annex**. Additional data and explanations. Click here for file
