# Supplementary material for: Remaining life expectancy among older people in a rural area of Vietnam: trends and socioeconomic inequalities during a period of multiple transitions
Source: BMC Public Health. 2009 Dec 17;9:471. doi: 10.1186/1471-2458-9-471 (PMC2803493; doi:10.1186/1471-2458-9-471)
Supplement: Additional file 1 — Annex. Additional data and explanations. [file 1471-2458-9-471-S1.DOC]

**Annex**


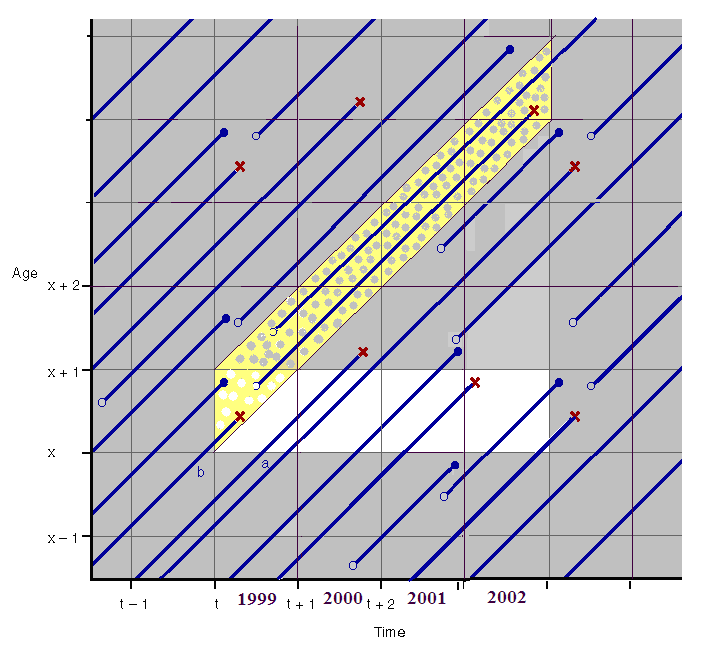


**Figure A1. A Lexis diagram on data used for calculating life expectancies**

The figure shows Lexis rectangle and Lexis parallelogram underlying period and cohort age-specific mortality rates, respectively.

**Table A1. Stepwise Cox regression concerning mortality of people aged 60 and over**

| Variables | B | SE | Wald | df | P | Exp(B) |
| --- | --- | --- | --- | --- | --- | --- |
| Period (2003-2006 vs. 1999-2002) | .470 | .058 | 65.990 | 1 | .000 | 1.599 |
| Male (vs. female) | .547 | .059 | 85.494 | 1 | .000 | 1.728 |
| Primary or less (vs. higher education) | .752 | .083 | 82.375 | 1 | .000 | 2.121 |
| Living with spouse (yes vs. no) | -2.127 | .064 | 1092.701 | 1 | .000 | .119 |
| Household head (yes vs. no) | .275 | .068 | 16.396 | 1 | .000 | 1.316 |
| Living with descendants (yes vs. no) | 1.712 | .077 | 498.778 | 1 | .000 | 5.538 |
| Poorest (vs. others) | -.385 | .087 | 19.514 | 1 | .000 | .680 |
| Poorer (vs. others) | -.155 | .074 | 4.368 | 1 | .037 | .856 |
| National poverty line (under vs. above) | 1.265 | .100 | 159.037 | 1 | .000 | 3.543 |
| International poverty line (under vs. above) | .409 | .085 | 23.370 | 1 | .000 | 1.506 |

**Table A2. Numbers of deaths and of person-years among people at age 60+**

**by periods and socioeconomic groups, 1999-2006**

| Sexes/ | Males | |  | Females | |
| --- | --- | --- | --- | --- | --- |
| Periods or groups | Deaths | P-Y |  | Deaths | P-Y |
| *Periods* |  |  |  |  |  |
| 1999-2002 | 327 | 7,636 |  | 352 | 13,196 |
| 2003-2006 | 344 | 8,300 |  | 376 | 14,125 |
| 1999-2006 | 671 | 15,941 |  | 728 | 27,331 |
| *Education* |  |  |  |  |  |
| Primary or less | 469 | 8,224 |  | 694 | 25,441 |
| Secondary or higher | 197 | 7,681 |  | 15 | 1,833 |
| *Household head status* |  |  |  |  |  |
| No | 499 | 4,513 |  | 584 | 8,258 |
| Yes | 172 | 11,428 |  | 144 | 19,072 |
| *Living with spouse* |  |  |  |  |  |
| No | 274 | 2,673 |  | 571 | 15,866 |
| Yes | 397 | 13,269 |  | 157 | 11,464 |
| *Living with descendants* |  |  |  |  |  |
| No | 121 | 2,575 |  | 107 | 4,831 |
| Yes | 469 | 10,049 |  | 551 | 17,487 |
| *House wealth quintiles* |  |  |  |  |  |
| Poorest | 108 | 1,616 |  | 130 | 4,967 |
| Poor | 145 | 2,640 |  | 133 | 4,981 |
| Middle | 135 | 3,417 |  | 164 | 5,601 |
| Rich | 148 | 4,558 |  | 166 | 6,649 |
| Richest | 135 | 3,711 |  | 135 | 5,133 |
| *National poverty line* |  |  |  |  |  |
| Under | 77 | 391 |  | 83 | 891 |
| Beyond | 594 | 15,551 |  | 645 | 26,439 |
| *International poverty line* |  |  |  |  |  |
| Under | 113 | 1,101 |  | 103 | 2,254 |
| Beyond | 558 | 14,840 |  | 625 | 25,076 |

**Table A3. Numbers of deaths and of person-years among people at age 60+**

**by socioeconomic groups, 1999-2002**

| Sexes/ | Males | |  | Females | |
| --- | --- | --- | --- | --- | --- |
| Periods or groups | Deaths | P-Y |  | Deaths | P-Y |
| *Education* |  |  |  |  |  |
| Primary or less | 244 | 4,369 |  | 329 | 12,575 |
| Secondary or higher | 70 | 3,233 |  | 5 | 573 |
| *Household head status* |  |  |  |  |  |
| No | 59 | 1,890 |  | 76 | 4,247 |
| Yes | 268 | 5,746 |  | 276 | 8,949 |
| *Living with spouse* |  |  |  |  |  |
| No | 240 | 2,411 |  | 251 | 3,548 |
| Yes | 87 | 5,225 |  | 101 | 9,648 |
| *Living with descendants* |  |  |  |  |  |
| No | 79 | 2,205 |  | 67 | 3,964 |
| Yes | 248 | 5,431 |  | 285 | 9,232 |
| *House wealth quintiles* |  |  |  |  |  |
| Poorest | 66 | 838 |  | 61 | 2,360 |
| Poor | 67 | 1,129 |  | 58 | 2,218 |
| Middle | 62 | 1,543 |  | 93 | 2,780 |
| Rich | 64 | 2,078 |  | 74 | 2,960 |
| Richest | 68 | 2,047 |  | 66 | 2,879 |
| *National poverty line* |  |  |  |  |  |
| Under | 60 | 270 |  | 64 | 512 |
| Beyond | 267 | 7,365 |  | 288 | 12,684 |
| *International poverty line* |  |  |  |  |  |
| Under | 79 | 631 |  | 73 | 1,166 |
| Beyond | 248 | 7,005 |  | 279 | 12,030 |

**Table A4. Numbers of deaths and of person-years among people at age 60+**

**by socioeconomic groups, 2003-2006**

| Sexes/ | Males | |  | Females | |
| --- | --- | --- | --- | --- | --- |
| Periods or groups | Deaths | P-Y |  | Deaths | P-Y |
| *Education* |  |  |  |  |  |
| Primary or less | 225 | 3,852 |  | 365 | 12,856 |
| Secondary or higher | 117 | 4,445 |  | 10 | 1,259 |
| *Household head status* |  |  |  |  |  |
| No | 76 | 1,848 |  | 79 | 4,405 |
| Yes | 268 | 6,452 |  | 297 | 9,720 |
| *Living with spouse* |  |  |  |  |  |
| No | 168 | 1,889 |  | 101 | 3,307 |
| Yes | 176 | 6,410 |  | 275 | 10,816 |
| *Living with descendants* |  |  |  |  |  |
| No | 99 | 2,665 |  | 69 | 4,445 |
| Yes | 245 | 5,634 |  | 307 | 9,680 |
| *House wealth quintiles* |  |  |  |  |  |
| Poorest | 52 | 879 |  | 73 | 2,580 |
| Poor | 73 | 1,413 |  | 75 | 2,675 |
| Middle | 81 | 1,988 |  | 78 | 3,176 |
| Rich | 75 | 2,151 |  | 87 | 3,141 |
| Richest | 63 | 1,869 |  | 63 | 2,553 |
| *National poverty line* |  |  |  |  |  |
| Under | 17 | 120 |  | 19 | 379 |
| Beyond | 327 | 8,180 |  | 357 | 13,746 |
| International poverty line |  |  |  |  |  |
| Under | 34 | 470 |  | 30 | 1,087 |
| Beyond | 310 | 7,830 |  | 346 | 13,038 |

**Table A5 - Percentages of people aged 60+ among general population**

**stratified by socioeconomic factors at four surveys, 1999-2006**

| Sexes/ | Male | | | |  | Female | | | |
| --- | --- | --- | --- | --- | --- | --- | --- | --- | --- |
| Groups | 1999 | 2001 | 2003 | 2005 |  | 1999 | 2001 | 2003 | 2005 |
| *Overall* | 7.2 | 7.7 | 7.8 | 8.0 |  | 12.9 | 13.4 | 13.7 | 14.0 |
| *Education* |  |  |  |  |  |  |  |  |  |
| Primary or less | 20.3 | 17.9 | 15.0 | 13.3 |  | 39.2 | 38.6 | 36.8 | 36.0 |
| Secondary or higher | 3.4 | 4.4 | 5.2 | 6.1 |  | 0.7 | 1.0 | 1.5 | 2.1 |
| *Household head status* |  |  |  |  |  |  |  |  |  |
| No | 1.9 | 2.1 | 2.2 | 2.3 |  | 4.1 | 4.4 | 4.6 | 4.7 |
| Yes | 19.6 | 20.6 | 21.2 | 21.2 |  | 53.9 | 54.8 | 55.1 | 54.7 |
| *Living with spouse* |  |  |  |  |  |  |  |  |  |
| No | 3.7 | 3.9 | 3.4 | 3.4 |  | 14.3 | 14.9 | 15.1 | 15.8 |
| Yes | 12.0 | 12.8 | 13.4 | 13.4 |  | 10.8 | 11.3 | 11.6 | 11.5 |
| *Living with descendants* |  |  |  |  |  |  |  |  |  |
| No | 2.4 | 3.0 | 3.1 | 3.5 |  | 5.2 | 6.2 | 6.0 | 6.7 |
| Yes | 17.4 | 17.5 | 18.0 | 17.6 |  | 25.1 | 25.0 | 26.0 | 25.5 |
| *Wealth index quintiles* |  |  |  |  |  |  |  |  |  |
| Poorest | 6.7 | 6.7 | 6.7 | 6.3 |  | 15.5 | 15.1 | 16.9 | 15.9 |
| Poorer | 5.9 | 6.5 | 7.2 | 7.3 |  | 12.0 | 11.8 | 13.3 | 13.4 |
| Middle | 7.0 | 7.5 | 7.9 | 8.0 |  | 12.2 | 13.3 | 13.1 | 13.6 |
| Richer | 8.4 | 8.6 | 8.4 | 8.8 |  | 13.3 | 14.1 | 12.9 | 13.8 |
| Richest | 8.2 | 8.9 | 8.7 | 9.1 |  | 12.3 | 13.3 | 13.1 | 13.2 |
| *National poverty line* |  |  |  |  |  |  |  |  |  |
| Under | 6.5 | 7.3 | 8.8 | 10.3 |  | 15.5 | 13.4 | 17.6 | 21.5 |
| Beyond | 7.4 | 7.9 | 7.7 | 7.9 |  | 12.0 | 13.5 | 12.9 | 13.3 |
| *International poverty line* |  |  |  |  |  |  |  |  |  |
| Under | 7.7 | 7.7 | 8.4 | 9.3 |  | 15.4 | 13.9 | 14.2 | 15.5 |
| Beyond | 7.0 | 7.8 | 7.4 | 7.5 |  | 11.9 | 13.0 | 13.2 | 13.2 |

**Table A6 - Distribution of people aged 60+ by socioeconomic groups**

**at surveys during 1999-2006**

| Sexes/  Periods or groups |  | 1999 | | |  | |  | 2001 | | |  |  | 2003 | | |  |  | 2005 | | |  |
| --- | --- | --- | --- | --- | --- | --- | --- | --- | --- | --- | --- | --- | --- | --- | --- | --- | --- | --- | --- | --- | --- |
|  | N | | % | 95%CI | | n | | | % | 95%CI | | n | | % | 95%CI | | n | | % | 95%CI | |
| *Male* | 1988 | | 36.1 | 34.8-37.4 | | 2048 | | | 37.0 | 35.7-38.3 | | 2090 | | 37.1 | 35.8-38.3 | | 2186 | | 36.9 | 35.6-38.1 | |
| *Female* | 3521 | | 63.9 | 62.6-65.2 | | 3487 | | | 63.0 | 61.7-64.3 | | 3547 | | 62.9 | 61.7-64.2 | | 3746 | | 63.1 | 61.9-64.4 | |
| *Male* |  | |  |  | |  | | |  |  | |  | |  |  | |  | |  |  | |
| Primary or less | 1245 | | 62.8 | 60.7-64.9 | | 1130 | | | 55.4 | 53.2-57.5 | | 1035 | | 49.5 | 47.4-51.7 | | 991 | | 45.4 | 43.3-47.4 | |
| Secondary or higher | 738 | | 37.2 | 35.1-39.3 | | 910 | | | 44.6 | 42.5-46.8 | | 1055 | | 50.5 | 48.3-52.6 | | 1194 | | 54.6 | 52.6-56.7 | |
| *Female* |  | |  |  | |  | | |  |  | |  | |  |  | |  | |  |  | |
| Primary or less | 3380 | | 96.7 | 96.1-97.3 | | 3313 | | | 95.3 | 94.6-96.0 | | 3281 | | 92.6 | 91.7-93.4 | | 3380 | | 90.4 | 89.4-91.3 | |
| Secondary or higher | 117 | | 3.3 | 2.7-3.9 | | 163 | | | 4.7 | 4.0-5.4 | | 264 | | 7.4 | 6.6-8.3 | | 360 | | 9.6 | 8.7-10.6 | |
| *Male* |  | |  |  | |  | | |  |  | |  | |  |  | |  | |  |  | |
| Household member | 363 | | 18.2 | 16.5-19.9 | | 387 | | | 18.9 | 17.2-20.6 | | 386 | | 18.5 | 16.8-20.1 | | 430 | | 19.7 | 18.0-21.3 | |
| Household head | 1635 | | 81.8 | 80.1-83.5 | | 1661 | | | 81.1 | 79.4-82.8 | | 1704 | | 81.5 | 79.9-83.2 | | 1756 | | 80.3 | 78.7-82.0 | |
| *Female* |  | |  |  | |  | | |  |  | |  | |  |  | |  | |  |  | |
| Household member | 956 | | 27.2 | 25.7-28.6 | | 961 | | | 27.6 | 26.1-29.0 | | 973 | | 27.4 | 26.0-28.9 | | 1019 | | 27.2 | 25.8-28.6 | |
| Household head | 2565 | | 72.8 | 71.4-74.3 | | 2526 | | | 72.4 | 71.0-73.9 | | 2574 | | 72.6 | 71.1-74.0 | | 2727 | | 72.8 | 71.4-74.2 | |
| *Male* |  | |  |  | |  | | |  |  | |  | |  |  | |  | |  |  | |
| Without spouse | 568 | | 28.4 | 26.5-30.4 | | 557 | | | 27.2 | 25.3-29.1 | | 456 | | 21.8 | 20.0-23.6 | | 485 | | 22.2 | 20.4-23.9 | |
| With spouse | 1430 | | 71.6 | 69.6-73.5 | | 1491 | | | 72.8 | 70.9-74.7 | | 1634 | | 78.2 | 76.4-80.0 | | 1701 | | 77.8 | 76.1-79.6 | |
| *Female* |  | |  |  | |  | | |  |  | |  | |  |  | |  | |  |  | |
| Without spouse | 2304 | | 65.4 | 63.9-67.0 | | 2232 | | | 64.0 | 62.4-65.6 | | 2258 | | 63.7 | 62.1-65.2 | | 2432 | | 64.9 | 63.4-66.5 | |
| With spouse | 1217 | | 34.6 | 33.0-36.1 | | 1255 | | | 36.0 | 34.4-37.6 | | 1289 | | 36.3 | 34.8-37.9 | | 1314 | | 35.1 | 33.5-36.6 | |
| *Male* |  | |  |  | |  | | |  |  | |  | |  |  | |  | |  |  | |
| Without descendants | 457 | | 22.9 | 21.0-24.7 | | 538 | | | 26.3 | 24.4-28.2 | | 548 | | 26.2 | 24.3-28.1 | | 630 | | 28.8 | 26.9-30.7 | |
| With descendants | 1541 | | 77.1 | 75.3-79.0 | | 1510 | | | 73.7 | 71.8-75.6 | | 1542 | | 73.8 | 71.9-75.7 | | 1556 | | 71.2 | 69.3-73.1 | |
| *Female* |  | |  |  | |  | | |  |  | |  | |  |  | |  | |  |  | |
| Without descendants | 867 | | 24.6 | 23.2-26.0 | | 958 | | | 27.5 | 26.0-29.0 | | 930 | | 26.2 | 24.8-27.7 | | 1079 | | 28.8 | 27.4-30.3 | |
| With descendants | 2654 | | 75.4 | 74.0-76.8 | | 2529 | | | 72.5 | 71.0-74.0 | | 2617 | | 73.8 | 72.3-75.2 | | 2667 | | 71.2 | 69.7-72.6 | |
| *Male* |  | |  |  | |  | | |  |  | |  | |  |  | |  | |  |  | |
| Poorest | 246 | | 12.5 | 11.0-13.9 | | 243 | | | 12.0 | 10.5-13.4 | | 256 | | 12.3 | 10.8-13.7 | | 260 | | 12.0 | 10.7-13.4 | |
| Poorer | 305 | | 15.5 | 13.9-17.1 | | 326 | | | 16.0 | 14.4-17.6 | | 349 | | 16.7 | 15.1-18.3 | | 394 | | 18.2 | 16.6-19.9 | |
| Middle | 393 | | 19.9 | 18.2-21.7 | | 421 | | | 20.7 | 18.9-22.5 | | 474 | | 22.7 | 20.9-24.5 | | 470 | | 21.7 | 20.0-23.5 | |
| Richer | 515 | | 26.1 | 24.2-28.1 | | 524 | | | 25.8 | 23.9-27.7 | | 497 | | 23.8 | 22.0-25.6 | | 516 | | 23.9 | 22.1-25.7 | |
| Richest | 513 | | 26.0 | 24.1-28.0 | | 519 | | | 25.5 | 23.6-27.4 | | 513 | | 24.6 | 22.7-26.4 | | 522 | | 24.1 | 22.3-25.9 | |
| *Female* |  | |  |  | |  | | |  |  | |  | |  |  | |  | |  |  | |
| Poorest | 686 | | 19.8 | 18.5-21.2 | | 629 | | | 18.2 | 16.9-19.5 | | 713 | | 20.1 | 18.8-21.4 | | 716 | | 19.5 | 18.2-20.8 | |
| Poorer | 607 | | 17.6 | 16.3-18.8 | | 599 | | | 17.4 | 16.1-18.6 | | 653 | | 18.4 | 17.1-19.7 | | 710 | | 19.3 | 18.0-20.6 | |
| Middle | 664 | | 19.2 | 17.9-20.5 | | 715 | | | 20.7 | 19.4-22.1 | | 729 | | 20.6 | 19.2-21.9 | | 772 | | 21.0 | 19.7-22.3 | |
| Richer | 759 | | 21.9 | 20.6-23.3 | | 796 | | | 23.1 | 21.7-24.5 | | 727 | | 20.5 | 19.2-21.8 | | 771 | | 21.0 | 19.7-22.3 | |
| Richest | 742 | | 21.5 | 20.1-22.8 | | 710 | | | 20.6 | 19.2-21.9 | | 723 | | 20.4 | 19.1-21.7 | | 705 | | 19.2 | 17.9-20.5 | |
| *Male* |  | |  |  | |  | | |  |  | |  | |  |  | |  | |  |  | |
| Under NPL | 394 | | 19.7 | 18.0-21.5 | | 475 | | | 23.2 | 21.4-25.0 | | 350 | | 16.7 | 15.1-18.3 | | 158 | | 7.2 | 6.1-8.3 | |
| Beyond NPL | 1604 | | 80.3 | 78.5-82.0 | | 1573 | | | 76.8 | 75.0-78.6 | | 1740 | | 83.3 | 81.7-84.9 | | 2028 | | 92.8 | 91.7-93.9 | |
| *Female* |  | |  |  | |  | | |  |  | |  | |  |  | |  | |  |  | |
| Under NPL | 992 | | 28.2 | 26.7-29.7 | | 940 | | | 27.0 | 25.5-28.4 | | 724 | | 20.4 | 19.1-21.7 | | 387 | | 10.3 | 9.4-11.3 | |
| Beyond NPL | 2529 | | 71.8 | 70.3-73.3 | | 2547 | | | 73.0 | 71.6-74.5 | | 2823 | | 79.6 | 78.3-80.9 | | 3359 | | 89.7 | 88.7-90.6 | |
| *Male* |  | |  |  | |  | | |  |  | |  | |  |  | |  | |  |  | |
| Under IPL | 552 | | 27.6 | 25.7-29.6 | | 1101 | | | 51.3 | 49.1-53.4 | | 1110 | | 53.1 | 51.0-55.2 | | 747 | | 34.2 | 32.2-36.2 | |
| Beyond IPL | 1446 | | 72.4 | 70.4-74.3 | | 1047 | | | 48.7 | 46.6-50.9 | | 980 | | 46.9 | 44.8-49.0 | | 1439 | | 65.8 | 63.8-67.8 | |
| *Female* |  | |  |  | |  | | |  |  | |  | |  |  | |  | |  |  | |
| Under IPL | 1156 | | 32.8 | 31.3-34.4 | | 1820 | | | 52.2 | 50.5-53.9 | | 1929 | | 54.4 | 52.7-56.0 | | 1286 | | 34.3 | 32.8-35.9 | |
| Beyond IPL | 2365 | | 67.2 | 65.6-68.7 | | 1667 | | | 47.8 | 46.1-49.5 | | 1618 | | 45.6 | 44.0-47.3 | | 2460 | | 65.7 | 64.1-67.2 | |

**Table A7. Values of T80/T60 calculated from data of FilaBavi and Sweden***

| Country & Period/Year | Male | Female | Both sexes |
| --- | --- | --- | --- |
| *Vietnam* |  |  |  |
| 1999 - 2002 | 0.09 | 0.17 | 0.14 |
| 2003 - 2006 | 0.12 | 0.21 | 0.18 |
| *Sweden* |  |  |  |
| 1979 | 0.12 | 0.16 | 0.14 |
| 1988 | 0.14 | 0.21 | 0.18 |
| 1999 | 0.18 | 0.26 | 0.22 |
| 2003 | 0.19 | 0.27 | 0.23 |

** Source of data for calculation*: Statistics Sweden: [Swedish Population (in one-year groups) 1860-2008](http://www.scb.se/Statistik/BE/BE0101/2008A01/Be01010Folkmängd1860-2008eng.xls). (http://www.scb.se/Pages/ProductTables25809.aspx)
